# Psychosocial Stress, Epileptic-Like Symptoms and Psychotic Experiences

**DOI:** 10.3389/fpsyg.2022.804628

**Published:** 2022-04-14

**Authors:** Petr Bob, Tereza Petraskova Touskova, Ondrej Pec, Jiri Raboch, Nash Boutros, Paul Lysaker

**Affiliations:** ^1^Center for Neuropsychiatric Research of Traumatic Stress, Department of Psychiatry and UHSL, First Faculty of Medicine, and Department of Psychiatry, Faculty of Medicine Pilsen, Charles University in Prague, Prague, Czechia; ^2^Rush University Medical Center, Chicago, IL, United States; ^3^Roudebush VA Medical Center and the Indiana University School of Medicine, Indianapolis, IN, United States

**Keywords:** stress, cortisol, epileptic like symptoms, psychosis, stress senzitization

## Abstract

**Background:**

Current research suggests that stressful life experiences and situations create a substantive effect in the development of the initial manifestations of psychotic disorders and may influence temporo-limbic epileptic-like activity manifesting as cognitive and affective seizure-like symptoms in non-epileptic conditions.

**Methods:**

The current study assessed trauma history, hair cortisol levels, epileptic-like manifestations and other psychopathological symptoms in 56 drug naive adult young women experiencing their initial occurrence of psychosis.

**Results:**

Hair cortisol levels among patients experiencing their initial episode of psychosis, were significantly correlated with stress symptoms measured by Trauma Symptom Checklist-40 (*r* = − 0.48, *p* < 0.01), and complex partial seizure-like symptoms measured by the Complex Partial Seizure-Like Symptoms Inventory (*r* = − 0.33, *p* < 0.05) and LSCL-33 (*r* = − 0.33, *p* < 0.05). Hair cortisol levels were not found to be significantly correlated with symptoms of anxiety and depression measured by Beck depression Inventory and Zung Anxiety Scale.

**Conclusion:**

These findings suggest a significant relationship between epileptic-like symptoms and stress responses demonstrated by patients in their first psychotic episode. These findings may suggest the potential for research to explore usefulness of anticonvulsant treatment in patients who do not respond to usual psychotropic medication.

## Background

Current evidence suggests that stressful early life experiences influence psychological and neurobiological development and ultimately have enduring consequences for the development of psychosis over the lifespan (Eack et al., 2008; van Os et al., 2008; van Winkel et al., 2008; Howes et al., 2017; Mayo et al., 2017). Further, recent research suggests that initial episodes of psychotic disorders may also be closely related to current stressful events (Varese et al., 2012; Reininghaus et al., 2016). As previously described, earlier or current stress may influence psychosis through its effects on the HPA axis (hypothalamic-pituitary adrenal) (Walker and Diforio, 1997; Faravelli et al., 2017; Pruessner et al., 2017)^8^ and cortisol reactivity more specifically (Ryan et al., 2004; Girshkin et al., 2014; Chaumette et al., 2016).

More recent research has suggested even more subtle ways through which early stressful and traumatic experiences may influence the brain and potential for psychosis. One of these involves the possibility that trauma may lead to sensitization or kindling-like processes which then become underlying mechanisms for seizure-like activity in schizophrenia and other psychiatric disorders (Roberts et al., 1992; Teicher et al., 2006; Bob et al., 2016; Weidenauer et al., 2017). Sensitization and kindling represent phenomena where repeated stimulations lead to a progressive enhancement of the response to repeated stimuli that may determine heightened vulnerability to epileptic seizures and increased sensitivity to stress stimuli (Goddard et al., 1969; Bertram, 2007). Further recent evidence also indicates that these kindling-like mechanism and sensitization may lead to neural processes resembling epilepsy which may manifest in patients with mental disorders including schizophrenia, posttraumatic stress disorder (PTSD), depression and others (Castner and Williams, 2007; Yuii et al., 2007; Collip et al., 2008; van Winkel et al., 2008).

Stress-related sensitization has additionally been suggested to create changes in GABA postsynaptic receptors that may lead to overstimulation of neurons mainly in the limbic system, resulting in limbic system irritability occurring as markedly increased prevalence of symptoms suggesting a subclinical form of temporal lobe epilepsy (Teicher et al., 2006; Bertram, 2007). These symptoms may manifest symptomatically similar to temporal lobe epilepsy including occurrence of somatic, sensory, behavioral and memory symptoms which may occur also in non-epileptic conditions (Roberts et al., 1992; Teicher et al., 2003; Gomes et al., 2016).

The issue of whether stress may lead to epileptic activity in persons with psychosis is an important issue for several reasons. For one it may point to the need for treatments which target seizure like activity and may also offer clues about treatment resistance among some patients (Johannessen Landmark, 2008; Tiihonen et al., 2009; Bob et al., 2010, 2011; Kaufman, 2011). It may further help us understand how psychosis when linked to trauma may follow a different course than psychosis not linked to trauma. To explore this issue this study has tested the hypothesis that cortisol levels among patients with first episode psychosis would be associated with both a more severe traumatic stress symptoms and heightened levels of seizure like activity. To measure cortisol we have analyzed hair samples. Heightened cortisol levels have been found in schizophrenia and bipolar disorders (Streit et al., 2016) using this method. The measurement of hair cortisol concentrations (HCC) represents new methodological approach and potentially provides relatively long-term balanced indicator of cortisol levels (Meyer and Novak, 2012; Stalder et al., 2017; Steudte-Schmiedgen et al., 2017) which may reflect chronic stress. To rule out the possibility that any of our observed results were the product of heightened levels of general anxiety or depression we included measures of these constructs as potential covariates.

## Materials and Methods

### Participants

The current study examines 56 adult women with whom the initial episode of psychosis was assessed immediately following admission to Psychiatric Hospital (mean of age 28.43, age range 20-38, SD = 5.32). All the patients were provided and assented to pre-informed consent at the onset of their hospitalization. Further, final written consent was obtained on remission, when the patients were able to deliver an informed decision. The study was approved by the University hospital ethical committee.

The subjects had predominantly high school education 14.35 (SD = 4.52) years. The subjects’ diagnoses of the initial psychotic episode was confirmed by clinical interview according to DSM IV guidelines and according to first clinical assessments fulfilled the criteria for any of the following diagnosis: schizophreniform disorder, schizophrenia, brief psychotic disorder, affective psychoses, schizoaffective disorder, and other psychoses. Patients were additionally assessed by M.I.N.I. version 5.0.0 (Sheehan et al., 1998). All the patients had their first hospitalizations and were administered no medications which influenced the CNS. Exclusion criteria were substance, and/or alcohol abuse, organic diseases involving the CNS, antiepileptic treatment, analgetic medication and benzodiazepine, or mental disabilities. Two of the authors of this article have performed independent re-evaluations of the patient’s diagnoses in accordance with DSM IV criteria (American Psychiatric Association, 1994).

### Psychometric Measures

The patients were assessed utilizing the Positive and Negative Symptoms Scale- PANSS (Kay et al., 1987) enabling evaluation of the typical positive and negative symptoms of schizophrenia (Cronbach’s alpha is 0.81 for positive symptoms and 0.88 for negative symptoms). The scale consists of 30 items divided into three subcategories: seven negative (PANSS-Neg), seven positive (PANSS-Pos), and 16 general (PANSS-Gen) psychopathological symptoms. Items may be rated from 1 (absent) to 7 (extremely present). Remission is typically defined as a score of <3 in all positive PANSS items.

Levels of experienced childhood trauma were evaluated with the TSC-40 (Trauma Symptom Checklist) (Elliott and Briere, 1992). The TSC-40 is a 40-item self-report style questionnaire utilizing a 4-point Likert scale. The TSC-40 evaluates symptomatology in adults associated with childhood or adult traumatic experiences and evaluates aspects of posttraumatic stress and other symptom clusters identified in some traumatized individuals.

Complex partial seizure-like symptoms were evaluated utilizing the complex partial seizure-like symptoms inventory– CPSI (Roberts et al., 1992). The CPSI was originally designed to evaluate sensory, somatic, behavioral and memory symptoms associated with temporal lobe epilepsy (i.e., brief hallucinations, dissociative disturbances, paroxysmal somatic disturbances, and automatisms). The inventory consists of 35 questions and subjects indicate the degree of their experience on a 6-point Likert scale (Cronbach’s alpha 0.95). Some recent evidence suggests a CPSI total score higher than 70 presents a significant criterion for the so-called epilepsy spectrum disorder although lower values also may indicate an underlying electrophysiological dysfunction (Roberts et al., 1992). Although these symptoms were originally described in patients with temporal lobe epilepsy, subsequent studies have found that transient sensory, cognitive, and affective phenomena occurring in patients with complex partial seizures may be more common in patients with affective disorders and also in other psychiatric diseases than is typically known (Silberman et al., 1985; Elliott and Briere, 1992).

The similar symptoms and experiences as those assessed by CPSI were also assessed by the Limbic System Checklist, LSCL-33 (Teicher et al., 1993). LSCL-33 is focused on evaluation and measurement of the temporo-limbic activity which may manifest as behavioral, sensory, memory and somatic symptoms which may include paroxysmal somatic disturbances, hallucinations and dissociative symptoms. Subjects indicate the degree of their experience on a four-point Likert scale (never, rarely, sometimes, often) (Cronbach’s alpha 0.90).

The assessment of depressive symptoms utilized the Beck depression inventory BDI-II (Beck et al., 1961, 1987) which consists of a 21-items questionnaire to indicate levels of experienced depression. Subjects indicate the degree to which their experience best corresponds to how he/she feels over the preceding 14 days on 4-point Likert scale.

The Zung Self-Rating Anxiety Scale was utilized to assess anxiety levels (Zung, 1971). The SAS is 20-item self-reporting inventory focused into the most common general anxiety symptoms. Each question is evaluated on 4-point Likert scale ranging from 1 to 4.

### Hair Cortisol Analysis

The hair samples utilized for biochemical assessment, were cut with clean scissors from the posterior vertex of the scalp due to the smallest hair variation in this position (Sauvé et al., 2007), stored at room temperature and sent in a sealed envelope. According to common procedures two 1 cm long hair segments were provided by each participant to assess ∼2 months of stress exposure, as hair grows at an average of 1 cm per month and there is a “wash-out” effect of cortisol from proximal to distal hair segments which enables to detect maximum of 3-6 month (Stalder et al., 2017). The analysis was performed on the average cortisol levels across the hair segments. Cortisol were evaluated utilizing an ELISA kit for cortisol in saliva (CORTISOL SALIVA ELISA, Diametra). The results were assessed utilizing photometric analysis ELISA (SPECTRA SLT) at the university biochemical department. This analysis of cortisol levels in hair was shown to provide valid and reliable results (Gow et al., 2010; Russell et al., 2012).

### Statistical Analysis

Statistical evaluation for results of psychometric measures included means and standard deviations. Because hair cortisol values do not have normal distribution we have used non-parametric Spearman correlation coefficients. We considered *p* < 0.05 as statistically significant. All the methods of statistical evaluation were performed using the software package Statistica version 6.

## Results

**FIGURE 1:**
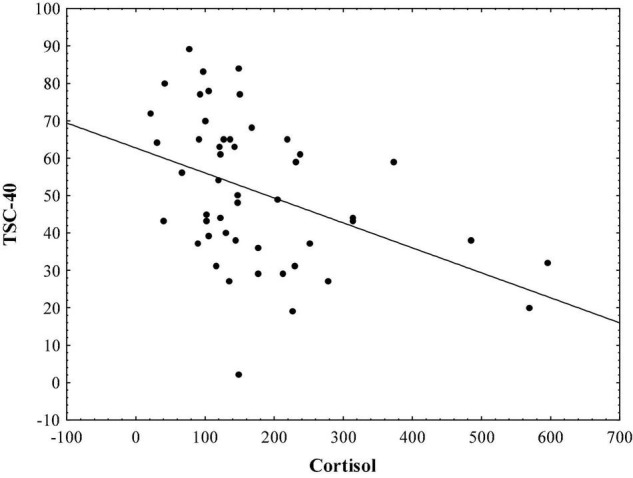
Relationship between hair cortisol levels (pg/mg) and stress symptoms measured by TSC-40.

**FIGURE 2:**
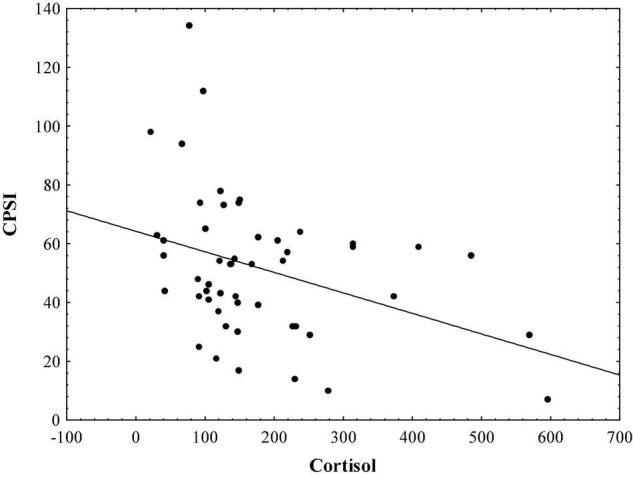
Relationship between hair cortisol levels (pg/mg) and epileptic-like symptoms measured by CPSI.

**FIGURE 3:**
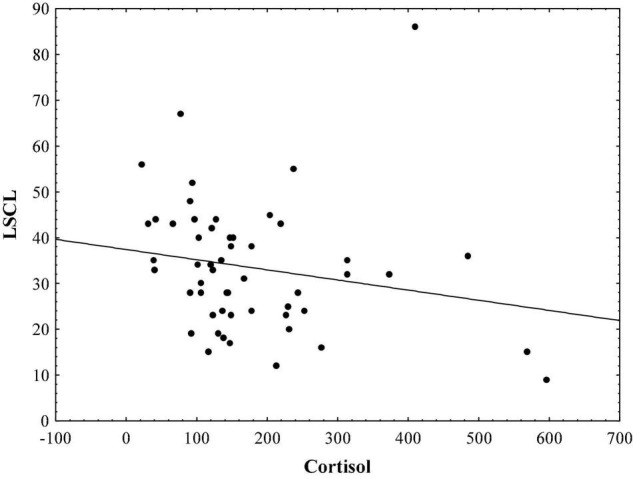
Relationship between hair cortisol levels (pg/mg) and epileptic-like symptoms measured by LSCL-33.

Results indicate that in the sample of initial psychotic episode onset subjects, hair cortisol levels are significantly correlated with stress symptoms measured by TSC-40 (*r* = − 0.48, *p* < 0.01; Figure 1), and also with complex partial seizure-like symptoms measured by CPSI (*r* = − 0.33, *p* < 0.05; Figure 2) and LSCL-33 (*r* = − 0.33, *p* < 0.05; Figure 3), but no significant correlations of hair cortisol were found with symptoms of anxiety and depression measured by SAS and BDI. Further the results suggest that complex partial seizure-like symptoms as evaluated by CPSI and LSCL-33 are correlated significantly with psychopathological symptoms related to depression, anxiety and stress. Traumatic stress symptoms evaluated by TSC-40 are significantly correlated with CPSI (*r* = 0.67, *p* < 0.01) and LSCL (*r* = 0.69, *p* < 0.01), symptoms of depression measured by BDI are significantly correlated with CPSI (*r* = 0.52, *p* < 0.01) and LSCL-33 (*r* = 0.42, *p* < 0.01). Symptoms of anxiety measured by SAS are significantly correlated with CPSI (*r* = 0.62, *p* < 0.01) and LSCL-33 (*r* = 0.80, *p* < 0.01). No significant correlations between symptoms of schizophrenia measured by PANSS subscales for Positive, Negative and Global psychopathology and other psychopathological symptoms related to stress, depression, anxiety, hair cortisol levels and epileptic like activity symptoms were identified.

From the 56 patients 8 had (14%) CPSI higher than 70, representing psychometrically reliable criterion for epilepsy spectrum disorders (Roberts et al., 1992; Bob, 2013).

## Discussion

The issue of whether stress may lead to epileptic activity in persons with psychosis seems to be supported by these findings suggesting underlying seizure like activity which may also offer clues about treatment resistance among some patients. In addition these findings may further help us to understand how psychosis when linked to traumatic stress may follow a different course than psychosis not linked to trauma. Results of this study provide first supportive indication as to the relationship between hair cortisol levels reflecting chronic stress conditions in patients with initial episode psychosis and epileptic-like symptoms.

These data based on the homogenous group of women reflect the drug naive mental and physiological states. This finding suggests “concomitant” correspondence of variables reflecting links between psychological experience and neurobiological changes related to stress. The results are in accordance with findings suggesting an influence of sensitization or “kindling-like” processes in etiology of psychiatric disorders such as drug addiction, schizophrenia, mood disorders, or posttraumatic stress disorder (Post and Weiss, 1996; Phillips et al., 1997; Post et al., 1997; Kraus, 2000; Collip et al., 2008; Weidenauer et al., 2017). The findings of the current study are also in agreement with evidence indicating that epileptic-like symptoms and mechanism of sensitization or kindling may be closely linked to stress conditions (Teicher et al., 2003, 2006; Yuii et al., 2007).

Some studies also indicate that similar sensitization or kindling-like processes may manifest in inhibitory systems due to local discharges of limbic and hypothalamic neurons and this dysbalance between excitation and inhibition leading to excessive inhibitory activities may manifest as psychotic disorders (Stephens et al., 2002, 2006). These patients who manifest the epileptic-like symptoms appear to be positively indicated for anticonvulsant treatment due to increased excitatory neural activity and regionally-specific compensatory upregulation of GABA-A receptors in response to decreased GABAergic input in hippocampal pyramidal cells (Heckers and Konradi, 2002; Mohler, 2006). The GABA neurons provide both inhibitory and disinhibitory modulation of cortical and hippocampal circuits and play an important role in gating of sensory information and attentional filtering within the corticolimbic system which are typically affected in schizophrenia (Glenthoj and Hemmingsen, 1997; Benes and Berretta, 2001; Costa et al., 2004; Gonzalez-Burgos and Lewis, 2008; Jacob et al., 2008). Further, the role of GABA neurons in cognitive functions suggests that disturbances in GABA systems may be linked to stressful conditions and alterations in the dopamine system (Benes and Berretta, 2001; Teicher et al., 2003, 2006; Yuii et al., 2007).

Numerous studies indicate that HCC is negatively related to hair washing frequency, hair treatment and oral contraceptive use, positively associated with ongoing chronic stress, some anthropometric measures, systolic blood pressure and with other confounding factors influencing hair cortisol such as gender, physical stressors and other psychiatric disorders (Meyer and Novak, 2012; Stalder et al., 2017). Some of these above mentioned variables were not addressed in this study, which represents a limitation with respect to more detailed analysis.

In summary, the current study suggests a link between epileptic-like symptoms and chronic stress response in the patients in the initial onset of psychosis. These data may be helpful in explaining the efficacy of anticonvulsant medication in patients who are resistant to usual psychotropic medication (Boutros et al., 2014). Patients with the epileptic-type manifestations likely may have decreased inhibition due to stress conditions manifesting as various mental and somatic states including complex partial seizure-like symptoms which may be assessed using psychometric measures (LSCL-33, CPSI). From the clinical perspective the link between complex partial seizure-like symptoms and stress may provide useful information for diagnostic consideration of anticonvulsant therapy and also how psychosis when linked to traumatic stress may follow a different course than psychosis not linked to trauma.

As for the limitations of this study the features of the sample limit the generalizability of these findings because the current data does not provide representative sample due to the number and gender of participants. Other limitations due to the novelty of this study do not allow to respond other interesting questions which this research might implicate. For example, the absence of significant correlations between symptoms of schizophrenia measured by PANSS subscales and symptoms related to stress, hair cortisol levels, and epileptic-like activity symptoms. Also further research focused on the efficacy of anticonvulsant medication in patients who are resistant to usual psychotropic medication their cognitive abilities and other psychopathological symptoms is warranted.

## Data Availability Statement

The raw data supporting the conclusions of this article will be made available by the authors, without undue reservation.

## Ethics Statement

The studies involving human participants were reviewed and approved by Charles University, First Faculty of Medicine Ethical Board. The patients/participants provided their written informed consent to participate in this study.

## Author Contributions

PB and TT: writing manuscript. PB, TT, OP, JR, NB, and PL: data analysis: PB, TT, and JR: data collection and processing. All authors contributed to the article and approved the submitted version.

## Conflict of Interest

The authors declare that the research was conducted in the absence of any commercial or financial relationships that could be construed as a potential conflict of interest.

## Publisher’s Note

All claims expressed in this article are solely those of the authors and do not necessarily represent those of their affiliated organizations, or those of the publisher, the editors and the reviewers. Any product that may be evaluated in this article, or claim that may be made by its manufacturer, is not guaranteed or endorsed by the publisher.
